# Facing the unknown COVID-19 pandemic: A qualitative study with nurses and nursing assistants in three European countries

**DOI:** 10.3389/fpubh.2022.985702

**Published:** 2022-11-29

**Authors:** Nertila Podgorica, Christoph Zenzmaier, Christine Rungg, Beatrice Bertini, Susanne Perkhofer

**Affiliations:** ^1^Health University of Applied Sciences Tyrol, Innsbruck, Austria; ^2^IRCCS San Donato Polyclinic, San Donato Milanese, Italy

**Keywords:** COVID-19, healthcare, nurse, nurse assistant, qualitative study

## Abstract

**Background:**

The coronavirus disease (COVID-19) is now a worldwide public health emergency. As essential and central parts of the COVID-19 patient care team, nurses and nurse assistants are facing all kinds of challenges caused by the disease and the pandemic. Understanding these challenges and the way nurses and nurse assistants handle and cope with them provides important knowledge on how to improve management of future pandemics and endemic situations. Thus, the present study explored the challenges faced by nurses and nurse asssitants who cared for COVID-19 patients in hospitals and long term care facilities in Italy, Austria and Germany.

**Methods:**

The study employed a qualitative design. Purposive sampling was used to select the participants consisting on nurses (*n* = 30), nurse coordinators (*n* = 6) and nurse assistants (*n* = 5) from hospitals (*n* = 32) and long-term care facilities (*n* = 9) in Austria, Germany, and Italy. Data were collected between August and December 2020 through semi-structured interviews. The collected data were analyzed using qualitative content analysis.

**Results:**

The analysis of the data revealed three main themes with twelve sub-categories: (i) Knowledge, skills, and training (lack of knowledge; skills; organizational issues; training); (ii) resources and risk (lack of protective equipment; difficulties with protective equipment; risk and infection; feelings and isolation); (iii) coping strategies (humor; adaption; team effort; self-care; family and friends).

**Conclusion:**

Nurses and nurse assistants who participated in this study faced many personal and professional challenges, and used different coping strategies to manage the situation. Some of these strategies can be applied to reduce these challenges and create better working conditions for nurses and nurse assistants in similar events. Further research, training of staff, and adaptation of institutional policies may help develop new strategies to face future pandemics successfully.

## Introduction

Coronavirus disease (COVID-19) is a respiratory infectious disease caused by a newly identified coronavirus named SARS-CoV-2. It emerged in China in December 2019 and has spread worldwide (1). The prevalence of COVID-19 disease is now a global public health issue. In March 2020, the World Health Organization declared the pandemic (2) COVID-19 disease outbreak as a new infectious disease currently has presented great challenges to the health systems of several countries (3). The disease has a high incidence rate in various countries around the world. Reports from the Worldometer database indicate that, for example, by 15 June 2022, the total number of confirmed COVID-19 cases was 541,881,388 individuals with a total of 6,334,516 deaths and 517,120,625 recovered patients in the world, with Europe having 199,227,238 cases and 1,848,747 deaths. Within Europe, Germany recorded 26,969,546 cases and 140,292 deaths, Italy 17,703,887 cases and 167,505 deaths, and Austria 4,305,432 cases and 18,715 deaths. Health professionals always play the most critical role in patient care during infectious disease outbreaks (4).

Pandemics negatively impact health systems and health professionals, who are the most important and leading force in the fight against pandemics (4). Moreover, nurses, the largest group of healthcare professionals and nurse assistants, work at the frontline of the healthcare system and provide care to patients with COVID-19 disease (5). In particular, nurses and nurse assistants provide direct patient care and learn from their experiences to better manage comparable future situations (6).

Health professionals, particularly nurses, met an unknown infectious disease, a daily changing situation due to new novel variants such as Delta and Omicron. Modifying treatment guidelines for this very contagious illness and caring for COVID-19 patients without knowing about the virus, treatment, and adequate care faced nurses and nurse assistants with different challenges (6, 7) Several studies have highlighted the various challenges that nurses experienced during this pandemic, such as being afraid of infecting their family members, stigma, restriction of personal independence, long and busy work hours, lack of high-quality personal protective equipment (PPE), lack of standard medical procedures, fear of severe emergencies in health, and fear of witnessing individuals and families grieving for their loved ones, which all negatively affect routine caring practices (4, 8–12). Furthermore, unstandardized PPE increased sweating and dehydration, and the feeling of suffocation from wearing masks for long periods, as well as the lack of proper food and drink conditions, could lead to a further physical burden on staff nurses during the COVID-19 pandemic (4, 13, 14). Previous studies demonstrated that nurses, during the COVID-19 pandemic, while facing different challenges, applied various coping strategies such as humor, teamwork, healthy eating, and communication with family and friends (8, 14–18).

The COVID-19 pandemic is a new challenge to worldwide healthcare systems and will not be the last one. Therefore, further exploration of healthcare professionals' experiences, particularly the general difficulties, feelings, and applied coping strategies of nurses and nurse assistants who have cared for COVID-19 patients in hospitals and long-term care facilities, will help to manage future crises better. Accordingly, this study aimed to examine the challenges faced by nurses and nurse assistants who cared for COVID-19 patients in hospitals and long-term care facilities during the first wave in Italy, Austria, and Germany.

## Materials and methods

### Design

A qualitative exploratory design using semi-structured interviews and qualitative content analysis was applied (19, 20). This design is appropriate for exploring personal experiences (20, 21).

### Ethical consideration

The study was conducted following the Declaration of Helsinki (20) and the Research Committee for Scientific and Ethical Questions of the Private University for Health Sciences, Medical Informatics and Technology (UMIT; Hall in Tyrol, Austria) and the Health University of Applied Sciences Tyrol (fhg; Innsbruck, Austria) approved the protocol (RCSEQ; Nr: 2767, Date 10.06.2020). The RCSEQ decided that the current research did not handle sensitive data from persons requiring special protection and patient data. All researchers were aware of the importance of protecting human subjects. Informed consent of the study participants was obtained in written form. Once the data was collected, the participants were anonymized using identifiers (P01–P41), and their names were removed from the transcribed data. The principal researcher kept the primary audio and video data in a secured file. The rest of the team worked with the anonymized transcripts. During the entire study, the participants were free to withdraw and request the deletion of their data at any time without any negative consequences.

### Sampling strategies and participants recruitment

This study was conducted in three neighboring European countries: Austria, Germany, and Italy. The inclusion criteria included registered nurses and nurse assistants working in hospitals or long-term care institutions who provided direct care to COVID-19 patients. Recruitment took place between August and December 2020 using purposive sampling that targeted nurses and nurse assistants who were known to the research team and fulfilled the inclusion criteria to benefit the most for this study (21). The participants were recruited via professional contacts of our international research team. Eligible participants were contacted by three female researchers (NP), (BB), and (CHR), who sent them information about the study, the socio-demographic questionnaire, and the informed consent *via* email, which was signed and sent back to the researchers who arranged Zoom meetings as suitable for the participants. Seventy participants expressed interest in participating voluntarily in this study, and ten withdrew due to personal issues. The recruitment and interviewing process happened simultaneously in all included countries. The sample size was determined based on data saturation, which refers to “the repetition of discovered information and confirmation of previously collected data” (22).

### Data collection

After reviewing relevant literature to obtain information regarding the experiences of the nurses and nurse assistants while giving care to COVID-19 patients, the authors developed an interview guide. Two Pilot interviews (not included in the analysis) were conducted to test the set guide. The interview procedure and questions were adapted per the pilot interviews. Data were collected through semi-structured in-depth interviews conducted online in the native language of the respective participant by a native-speaking author between August and December 2020. The final interview guide covered various aspects of the COVID-19 pandemic. Questions of the interview guide related to the data presented in this paper are shown in Table 1.

**Table 1:** Questions from the interview guide.

**Interview questions**
Could you tell me about your work experience as a nurse/nurse assistant?
Probe: education, workplace, training, and general information
Could you tell me about your experience and preparation before caring for a patient with COVID-19?
Probe: preparation, performing procedures, communication
Could you tell me what challenges did you experience when providing care for COVID-19 patients?
Probe: barriers, challenges. Could you expand on that? Can you give me an example? How did you overcome these challenges?
Could you describe your feelings related to this situation? How did you manage to continue working in such a situation?
Probe: feelings, coping strategies. Could you expand on that? Can you give me an example? How did that help you?
Before closing the interview: Is there anything else you would like to tell me?

Due to the high transmission risk of COVID-19 and visiting restrictions in the institutions, video methods *via* Zoom were preferred. Each participant received a private email with an invitation to the interview and information about the aim and content of the study. Furthermore, they got information about the research team before arranging the interview. The date and time scheduled for the interview were agreed upon by both the participants and the interviewer. After receiving participant's written and oral permission, all interviews were conducted and audio and video-recorded. During the interviews, it was ensured that both the participant and the interviewer were alone and in a quiet environment. All interviews were immediately transcribed verbatim using F4 software (23). The interviews were conducted, transcribed, and analyzed by authors who are Italian and German native speakers, respectively. Interviews lasted for an average of 50–70 min. and were terminated when the participant had no additional points to state. The interviewing process continued until saturation was reached in the interviews obtained in each country (20, 21) in a total of 41 interviews (Table 2). In addition, the researchers also took field notes during the interviewing process (24). Field notes provide insights into the participants' behavior and all the expressions and events during the interview process (25). Finally, a socio-demographic questionnaire was created to collect the participant's characteristics (Table 2).

**Table 2:** Participants sociodemographics.

Participants [*N*]	41	
**Country [*N* (%)]**		
Austria	10	(24.4%)
Germany	8	(1.5%)
Italy	23	(56.1%)
**Sex [*N* (%)]**		
Female	31	(75.6%)
Male	10	(24.4%)
Age, years [mean (range)]	37.1	(24–60)
**Educational level [*N* (%)]**		
Nurse, BScN	30	(73.2%)
Nurse Coordinator	6	(14.6%)
Nurse-assistant	5	(12.2%)
Working experience, years [mean (range)]	12.5	(1–36)
Work extent in % during pandemic [mean (range)]	89	(35–100)
**Working place during pandemic [*N* (%)]**		
Hospital	32	(78.0%)
Long-term care	9	(22.0%)
**Any differences in daily working [*N* (%)]**		
Yes-Transfer to another station	3	(7.3%)
Yes-Different shifts	4	(9.8%)
Yes-overtime	16	(39.0%)
No	18	(43.9%)
Number of beds in department [mean (range)]	25.1	(8–50)
Number of COVID-19 patients cared for [mean (range)]	34.7	(4–100)
**Previous training on pandemics [*N* (%)]**		
Yes	5	(12.2%)
No	36	(87.8%)
**Working instructions before starting at COVID-19 department [*N* (%)]**		
Yes	23	(56.1%)
No	18	(43.9%)
**Protective equipment [*N* (%)]**		
Sufficient	32	(78.0%)
Insufficient	9	(22.0%)
Number of people living with [mean (range)]	1.9	(0–4)
**Spiritual / religious person [*N* (%)]**		
Yes	23	(56.1%)
No	13	(31.7%)

### Data analysis

The video recordings were immediately transcribed verbatim and reviewed by the authors who speak the respective original language and English for accuracy using spot-checking, taking a small set of transcripts (4 of 41), and reading and rereading the transcripts and the field notes (26). Afterward, the transcripts were translated into English following Santos et al. (27) strategy for translating qualitative interviews and research reports. They suggest having language experts on research teams and translation and back-translation issues directly considered within the team. This strategy reinforced the approach of Al-Amer et al. (28), who recommend carefully selecting bilingual and bicultural team members with sufficient qualitative research expertise and using translators with similar cultures, languages, and discipline expertise. Data analysis was conducted by (NP), (BB), (CHR), and (CHZ) through Mayring's qualitative content analysis method (19, 22).

The directed content analysis combined deductive and inductive strategies (20, 22). Further work on qualitative analysis was considered to achieve a multifaceted view of this method and implemented with the qualitative data analysis software MAXQDA 2020 (29). To begin, a codebook, which defined initial codes, was developed based on the research questions and the previous literature, which motivated the study. The four team members who did the initial, open coding of the data also added codes inductively as new themes emerged from the interviews. The second round of focused coding was done, which integrated the new codes and identified connections between codes. In a third round, all the codes were, subsumed into subcategories and categories (Table 2). In the final step, the research team discussed discrepancies in coding (19, 29).

### Validity, reliability/rigor, and trustworthiness

This study was conducted according to the consolidated criteria for reporting qualitative research (30) and the standards for reporting qualitative research (31). Results and conclusions are robust since it is the analysis of 41 interviews, and the strategies such as trustworthiness, credibility, transferability, dependability, and conformability of data were duly considered. The study achieved credibility *via* in-depth interviews followed by adding, checking, and approving the extracted codes. Consistent with guidance from Elo et al. (32), data were organized systematically, with more than one investigator involved in coding and analysis. Four authors independently analyzed the transcripts. The findings were then contrasted and conversed until consensus on categories, subcategories, and codes were achieved. Transferability was accomplished by considering differences in the interviewees' characteristics and adequate quotes acquired through interviews. Dependability and conformability of the results were also ensured through reporting and recording of the study steps and processes (32).

## Results

Forty-one interviews were analyzed to describe the nurses' and nursing asisstants' challenges in the context of COVID-19 care. The socio-demographic data is described in Table 2.

In the course of the first wave of the pandemic, nurses and nurse assistants in Italy, Austria, and Germany faced unpredictable emergent situations that were summarized in three major themes (i) knowledge, skills, and training, (ii) resources and risks, and (iii) coping strategies (Table 3).

**Table 3:** Categories and subcategories.

**Category**	**Subcategory**
Knowledge, skills, and training	Uncertainty and insufficient preparation
	Skills, organizational issues
	Training
Resources and risks for the	Lack of protective equipment
healthcare professionals	Difficulties with personal protective equipment
	Risk and infection
	Different feelings and isolation
Coping strategies	Humor
	Adaption
	Team effort
	Self-care
	Family and friends

### Knowledge, skills, and training

#### Uncertainty and insufficient preparation

At the beginning of the COVID-19 outbreak in Europe, there was limited knowledge about the virus, the disease, infection, protection, and control. Most participants from Italy said they felt uncertain and unprepared for such a pandemic. They compared the COVID-19 pandemic to a tsunami because the situation worsened quickly. Healthcare professionals and corporate bodies were unprepared to take the necessary measures and acquire the knowledge needed to deal with such a situation:

“*It was an unforeseeable thing, in the sense that even... especially at the beginning, no one was prepared for this virus, no one knew about it, and therefore treating any patient was difficult at the beginning.” (I-P4)*

“*In this period, when this “new job” arrived for us, it was like a tsunami because we had to reorganize ourselves mentally and personally in managing a new situation to which we were not accustomed.” (I-P5)*

Some participants from Austria and Germany, where the first pandemic wave came later, reported specific preparation measures such as reorganizing wards to improve intensive care capacities, purchasing orders for protective equipment, or implementing internal hygiene guidelines and protocols. However, several participants, mainly from nursing homes, felt unprepared and did not experience preparative measures in their facilities.

“*We had a training session exactly 1 day before the ward (.) was converted to a COVID ward (.). A lady came from (...) I don't remember which department, but she did the training with us. Yes, how to properly enter and exit. The handling, how to do. The communication with the patients...” (A-P32)*

“*Okay, so in the first wave, which started at the end of March, I work in an intensive care unit with ten beds. Since February, we have had four beds constantly blocked, and to prepare for the wave.” (G-P35)*

“*You could have used the time to prepare better. I think things didn't go so well at my ward. My ward manager didn't take it that seriously. And the staff were all very concerned. I mean, it was new for everyone, but in the hospital, it was just again different. And um, there was no training, no briefing, but “so please do it now.”” (G-P38)*

#### Skills, organizational issues

Almost all interviewees reported a lack of practical skills in treating COVID-19 patients. Patients overloaded hospital capacities by far, and many died in a short time after being admitted to the hospital, providing no time for any treatment. In addition, some Italian interviewees indicated that they lacked time to learn about COVID-19 and new practical skills during the first wave of the pandemic. Furthermore, some interviewees talked about organizational problems related to the lack of information and skills:

“*Unfortunately, there was no time to get information because there was no information available, so we launched into this “fight” with our eyes closed, without knowing exactly where we were going.” (I-P7)*

“*We didn't have a standard working technique because everyone came from different departments. I usually work in general medicine, but I worked with people from psychiatry or a pneumology ward, so there was a bit of organizational difficulty from the team's point of view.” (I-P12)*

At the time of the interviews (around the second wave of the pandemic and still before vaccination was available), participants complained about a lack of clear evidence-based protocols and safe treatment.

“*What kind of treatment could we provide for COVID-19 cases when we are living with his situation for 9 months, and we still don't know what/a safe cure or vaccine is not yet known (.), and we're still as in the first day even though 8–9 months have passed?” (G-P40)*

#### Training

While some interviewees talked about the unexpected situation they faced, they pointed out that they had undergone brief training on preparedness very soon after the pandemic started. Such training mainly focused on using protective equipment, hygiene, and infection prevention, mainly consisting of online (video) lessons.

“*The only course I did was one online course on protective equipment, so let's say regarding what had to be done legally precisely and what had to be done because it was a rule. Regarding courses on managing the COVID patient, they were not done. Probably, there wasn't even the time, but the courses weren't done. I learned everything on the field.” (I-P11)*

“*Some training courses were done, but they were more about staff protection, hygiene protection, and transmission protection. These were the training that was mainly offered.” (G-P40)*

In the first phase of the pandemic, effective practical training for protective materials was frequently impossible due to material shortages. Only a few participants received training and instructions on-site at their facility. Some participants wished for more advanced training on treatment options, such as training prone positioning.

“*What I found burdensome, really burdensome, was that I thought: There are so many recommendations from the professional associations (...), and this is not being implemented. Or it takes so long. Or, of course, we don't train the prone position. (...) We don't train how to put on and take off protective clothing. Because there is not enough.” (A-P25)*

Some participants indicated that they learned many new techniques during the COVID-19 pandemic. They described their experience during the COVID-19 pandemic as unique because they learned to use protective equipment and all the necessary devices for an emergency of this nature and how to manage patients in such critical situations. One of the Italian participants said that they learned everything by doing.

“*Everything we learned in the field, in the COVID phase. Just 1 day before we opened the department, our managers gave us a course on dressing and undressing and talked to us in a little more detail about COVID and the studies that had just emerged, but it was all speedy. What we learned, we learned in the field, mainly.” (I-P9)*

Participants also helped by sharing their knowledge and teaching other colleagues the techniques they knew. The formation of new teams by successfully integrating colleagues from other wards or agency workers facilitated learning from each other.

“*So, we looked for videos, we asked for information (...), we structured ourselves in a little way / We gave ourselves tasks so that everyone looked for indications on how to dress (...), that is, we watched the videos, and we did rehearsals. I did many trials on how to dress and undress; I did a lot of them. And then I showed it to the others.” (I-P23)*

“*Yes, the teamwork and the solidarity in the team was excellent (.) and (.) because we also had so many temporary workers, (.) so they are incredibly well trained, and I learned a lot from them in practice, as far as ventilation is concerned and (.) all kinds of things.” (A-P33)*

On the other hand, some wards reported a lack of communication and information within the team.

“*The chief physicians have constantly communicated with us in the intensive care unit and have exchanged ideas. Unfortunately, not so intensively with the nursing team, but only the heads, yes, and the communication has fallen by the wayside a bit, which I found very unfortunate.” (G-P35)*

Participants reported that constant learning during the pandemic and successful adaptation of treatment protocols positively affected professional self-confidence and job satisfaction.

“*Um (....) it's a constant learn (...), you go into work already self-confident. Therefore, with specific experience values, you go into the situation in (...) relatively more relaxed. If a patient comes from the shock room, you go into the matter completely different because you have the experience.” (A-P24)*

“*Well, it gives me joy. I also enjoy my job more. I'm learning; that gives me pleasure and this incredible good teamwork.” (G-P38)*

### Resources and risks for the healthcare professionals

#### Lack of protective equipment

Participants generally perceived a lack of resources regarding personal protection in the workplace. There was an initial lack of equipment and other resources, which posed a greater danger to health care workers and the patients they were in contact with. The most common complaints included the lack of masks, gloves, hand gels (hydro alcoholic solution), demand for proper use, and the establishment of collective protection measures:

“*Another problem did not have the protective material. You can't arrive on Monday to count the masks to wear to be able to enter an intensive care unit with COVID and say, “Until Thursday, we have some; after that, we don't know if they'll arrive.” When you experience them first hand, these are things that you say, “Jesus, how have we ended up?” These things are worrying (...), because none of us was willing to go in without protection because it was like going to the slaughterhouse; I mean, what do you do? Do all go in like this, without protection? That doesn't exist, that is/ Afterwards, the problem of not having enough protective material existed probably all over the world because it was also seen in America and other places. Still, in my opinion (...) it had to make people think and act accordingly to what the situation was, that is.” (I-P19)*

“*We didn't have enough masks, for example. FFP2 masks and FFP3 masks, we always had to save. Um, and we were also controlled. Of course, that also destroys the trust relationship a bit (...). And then everyone looks a little bit, well ok, but now I have to protect myself. And not to show any consideration for the others.” (G-P38)*

#### Difficulties with personal protective equipment

While many participants reported a lack of resources and protective equipment, sufficient protective material was initially available in some facilities, particularly in Germany and Austria. However, lack of knowledge, training, and insecurities are sometimes misused.

“*I can say that the equipment was misused a lot due to the lack of knowledge (..) of the virus, or how can I call it? The difficulty or the severity of the virus; then, the equipment was highly misused. Though, over time it was realized that resources were running out.” (G-P40)*

Some participants talked about the difficulties they faced while working with the protective equipment due to physical problems such as exhaustion caused by heat and breathing problems or because they could not touch the patients, couldn't speak to them, or keep eye contact.

“*We were not used to working with (..) let's say, FFP2 masks, which gave an intense headache. At the end of my shift, I had a headache related to using the visor.” (I-P12)*

“*The mask's loops are as unbearable as they cause ear irritation and “decubitus,” sometimes they become red. We are under time pressure and stressed, which is even more problematic (touching the ears). The air is limited (.) in some way.” (G-P41)*

Participants frequently mentioned the additional workload and burden caused by dressing and changing protective equipment.

“*We should wear (.) shoe covers over the shoes before entering the room; we enter, and/we should change the clothes every time we enter and get out of a room. So, entering a room, getting out, and entering another room. It's not that/we cannot enter a room with the same clothes. We should change clothes, and wear new ones. So, it's the same thing throughout the day.” (G-P41)*

Several participants also perceived patients or residents as distressed when nurses were suddenly dressed in complete protective equipment.

“*I would say that the patients*' *reaction when they resulted positive (.) was bad, and they were uncomfortable when the personnel got dressed up like that.” (G-P40)*

“*That was also the problem because the (.) older, demented patients thought we were aliens. (…) I don't know what they thought, but they panicked, and we had to sedate one of them because (.) she went crazy. And I understood that” (A-P29)*

#### Risk and infection

Because nurses and nurse assistants faced a previously unfamiliar emergency, most of them initially did not know how to protect themselves and others with absolute certainty. As they saw the situation worsen and colleagues getting ill or dying, they started to self-isolate, fearing infecting themselves or spreading the virus to family members.

“*When I saw some colleagues falling ill, both doctors and nurses, I decided to move away. I went to live in a flat alone, quite close to my family so that I could see them from the terrace, but I lived in a flat alone because I was afraid of infecting them.” (I-P18)*

While deaths increased daily, healthcare professionals did not know how to treat patients and their sickened colleagues and felt at higher risk. With tears in their eyes, some interviewees stated that they felt frightened and in danger of their lives, their family's, and their colleagues' lives.

“*Watching my colleague die (cries) and choosing whether to intubate him or not. That was the most painful thing. I hope I'll never have to live it again, never again! I hope I will never feel that again. I don't want to see a colleague of mine suffocate, to hear a colleague of mine telling his wife, “This is the last time I'm going to say goodbye to you because I have to be intubated if I want to live, but unfortunately I know I'm never going back.” (I-P8)*

“*I also had a relatively young colleague, 26, and she was very sick. So, she came to work after 3 weeks and was extremely dyspnoeic. You couldn't talk to her normally, so she was breathing calmly, which got me. Before that, I was like, “yeah, okay, Corona is already bad,” so I also felt it was bad, but I thought like, “well, if you get it, you're young, it'll be okay somehow” and then when I saw that, it already scared me. You don't know what the consequences will be. You don't know how it will happen.” (G-P36)*

Carelessness and negligence were frequently associated with infection among healthcare professionals. In some cases, interviewees working in nursing homes stated that some of their colleagues did not wear masks or did not follow the appropriate rules and were infected by patients. In other cases, they were the ones who infected patients without knowing they were COVID-19 positive. Some participants stated that most nursing home residents were infected and, therefore, all staff was infected. In other cases, although some colleagues tested COVID-19 positive, they continued to work.

“*Because (.) yes, so you don't feel well and (..) yes. I've also seen colleagues who didn't take hygiene very seriously, sitting in bed with patients in a protective gown, and I thought, that's impossible, that he wouldn't get infected.” (A-P33)*

“*We had a colleague, who saw it very (.) um (.) chillingly, so very relaxed, then came out with the mask, with which she was in the patient room, and afterward she came in the nursing office and hung the mask down over the table. then a colleague asked me during the check-in (.) “Is this the mask that she was wearing with the patient?” “Yes, and?.” So we didn't perceive the seriousness of the situation at all at the beginning.” (G-P35)*

Several organizational problems arose due to many infections among healthcare professionals. The condition of healthcare professionals led to a personnel shortage, resulting in the nurses having to deal with various difficulties.

“*There are personnel shortages, especially in the wards where there are mainly COVID patients because a part of them have resulted positive, unfortunately; some others are not present because of being sick or other reasons. So, (..) it's difficult, at the moment. There are shortages.” (G-P39)*

Some Italian participants reported that the situation became even more complicated when the head nurses became sick.

“*We were alone without the head nurse because she also fell ill immediately due to COVID-19. We did not need management, but we had already needed a guideline. Then, one of our colleagues died, and we saw his death until the end. So, practically speaking, we lacked organization and the possibility of working well with available management. Then, organizational, yes, because (...) people got sick; we remained very few at work and were always the same staff.” (I-P8)*

Other interviewees stated a shortage of tampons and treatment for their infected colleagues. It led to a chaotic situation and changed the work rhythm of the healthcare professionals. Since most of the healthcare staff was ill, the nurses and their assistants who were still healthy had to work extended shifts every day and cancel their yearly vacations.

“*Yes, as far as shifts are concerned, I had a much more irregular rotation in the previous period, from March 1*^*st*^ *to March 17*^*th*^*, where many colleagues fell ill. Therefore, we were all asked to skip the rest periods, return, and extend, and it was crazy with the shifts, but we followed a regular rotation. (...) It happened once that a colleague had tested/ when they started to do the serological tests, she tested positive for the serological test, so clearly, she did the swab, and while waiting for the swab report, she couldn't come to work. Therefore I had to skip a rest to come back, but not/ other times not, the shifts were regular.” (I-P11)*

#### Different feelings and isolation

Several participants experienced feelings of stress, anxiety, and fear facing COVID-19. They did not know how to protect themselves and feared contracting their families the virus.

“*Well, we even had to wear; I once had to wear glasses, diving goggles. Because at that time, we didn't know how it would be transmitted, and somehow I took everything that was available in terms of protective equipment, even though I now think to myself, how is this supposed to get through my glasses into my eye? (...) But of course, I was still in a situation where I was even more afraid than I am now.” (A-P29)*

“*A world pandemic which caused stress for the medical and nursing staff. Every time we entered that room, we had to be isolated with masks and PPE, and it was a huge emotional load.” (G-P40)*

“*The nurse mothers were terrified of bringing the virus home to their children, but it was the same for me, for my mother who is an elderly, as it was unknown.” (I-P2)*

“*I have never been afraid that I would infect myself. I've only always been afraid that I'd carry it into the home. Because that would be fatal, of course.” (A-P27)*

Although healthcare professionals had already begun implementing protective measures for safety and hygiene (based on previous experience with pandemics), participants stated they began to isolate themselves to protect their families.

“*When I saw some colleagues falling ill, both doctors and nurses, I decided to move away. I went to live in a flat alone, quite close to my family so that I could see them from the terrace, but I lived in an apartment alone because I was afraid of infecting them.” (I-P18)*

### Coping strategies

When participants in this study were asked how they coped with the situation, they indicated that they applied various strategies to cope with the actual situation. Some of the individual methods nurses and nursing assistants used are explained below.

#### Humor

Many participants used humor in their professional teams or families as a strategy to cope with stress, fear, panic, and drama caused by the pandemic. They showed that even in such a tragedy, there could be many humorous situations from which one learns how to deal with difficult situations in life, give and receive more love, and add the color of positivism to this unique experience.

“*Well (..), maybe when we were joking together or/ (..) Inside, all locked up, we were joking together, almost as if it was nothing. So, it was nice, because it means that we love each other, because (laughs) if in times of difficulty.” (I-P22)*

Giving a smile or creating humorous situations has proven helpful for healthcare professionals and patients.

“*So even in the ward, maybe we tried to “laugh” with the patient who maybe couldn't hear us because he had the ventilator, so, maybe we made some gestures; anyway, in this tragedy, we always tried to make the persons smile.” (I-P4)*

Several participants reported a grim sense of humor between colleagues due to stressful work.

“*Yes, that is a bit of gallows humor. Meanwhile, it is like this, if someone coughs at our ward, we don't say “Bless you!” anymore but “COVID!” so (laughs) we have developed peculiar and unusual humor, but I think that in a special area, it is an outlet. As long as it's not offensive or you still know what it's about, I think it's within reason.” (A-P24)*

#### Adaptation

Our study participants indicated that they learned to live with and adapt to the actual situation during the pandemic. At the pandemic's beginning, specific knowledge was limited, but as the days passed, they studied and acquired expertise about COVID-19 and learned how to behave and act.

“*Then, well, the fear of contracting a disease that no one knew about; therefore, we didn't know how to deal with it. So we studied the disease daily, checked the internet, and updated ourselves; it was a unique update to face this crisis safely.” (I-P23)*

“*Actually, all established concepts. These guiding principles, everything we did was somehow all (...) so void because it came differently every day. In addition, you can't stop it anymore, so we said it doesn't matter how we do it. The main thing is that they are cared for well, and we are fine.” (A-P26)*

Nurses and nurse assistants had to adapt to the situation, using protective equipment, isolation, insults, or a lack of equipment and medication. Some participants said their team missed this time because they learned to work under these conditions and difficulties, which also had a positive aspect.

“*So, it wasn't easy. However, we adapted, we understood that we had to change our habits. Anyway, we keep on doing it, and I hope that we have the strength to continue.” (I-P5)*

Several participants shared their opinion that COVID-19 will continue being a health care issue and that everyday life has permanently changed, at least for the next couple of years. They got used to protective measures such as masks and routine testing for infection at work and in everyday life. This adaption to the pandemic led to reduced perceived risk. Some participants stated that they are no longer afraid of being infected, while others even reported careless use of protective measures.

“*Some colleagues still have such panic (.) yes, and there are employees who are more relaxed now. So I have to say, I am now/. I would no longer be afraid of getting infected, and then I just wear protective equipment.” (A-P31)*

“*Yes, and I think they were also more aware of their material, so they dealt with it more consciously. Such things like ““oh, come on, it ‘'s okay”” or ““I”ll just have a quick look here,”” or ““I don't need any protective equipment for that,”” or something like that. That was not the case in the first wave.” (G-P38)*

#### Team effort

Different ways of coping with the COVID-19 pandemic were of great interest to the participants in this study. Conversations, hugs, despair, and support among friends and colleagues were successful coping strategies.

“*Well (...), actually talking with colleagues, because by experiencing the same situation, we got (..) strong. Let's say that living the same situation we also (laughs)/ We said, “Wow, but what are we living?” (laughs). But we got strong together.” (I-P17)*

“*So I think it would be more difficult if I didn't talk about it with my friends. I have many friends who work in the health care system so that I could talk about it very well with them and quasi so yourself (.) experience a bit (.) of pseudo-psychological care, and then it goes already.” (G-P34)*

The team support helped many of the participants to handle the demanding situations.

“*Two colleagues were about to collapse and maybe even cried during their shifts for several days. We tried to make a team effort and said: “Come on, if you can't do it, stay at home, but if you stay here, move on, because if you cry, then I cry, we cry in two, it becomes a disaster.” Then, they managed to do it.” (I-P4)*

Another participant stated that he managed to cope with the situation by using organizational strategies for himself and the team in which he worked, taking on the role of teacher for some young colleagues, and avoiding anxiety and panic situations.

“*At the beginning, it was more a handing over of tasks among colleagues, among more experienced colleagues or those who had already (...) done shifts in those departments and explained to the others how to dress and undress.” (I-P18)*

#### Self-care

Several participants indicated that it was crucial to protect themselves physically and mentally; otherwise, they would have gone crazy. They showed that they tried to follow all hygiene rules, not shake hands or hug each other, support each other emotionally, and work as a team. They joined and tried to eat well and strengthen their immune systems.

“*Also, do something for myself and (...) for the physical I did a detox treatment with an energy specialist. So much for the spiritual topic, Yes, I believe in something like that. So I went to the energy specialist, and this body's detoxification was good for me.” (A-P26)*

“*Self-protection and protection of the other (.), also protection for my family of course.” (G-P35)*

*And then, I would advise them [young colleagues] to try to detach their mind when they leave the hospital and (...) not to take home too many, let's say, psychological burdens from there, to try to keep the working life separated from everyday life.” (I-P16)*

#### Family and friends

Most of the participants said that they managed to cope, work and succeed in the pandemic situation only thanks to the support and love of their families.

“*And then, they practically facilitated me in everything at home, and I didn't do anything. I practically went to work, arrived home, and maybe slept in the afternoon. I only washed my clothes, and that's all. I made my bed; I did my things, I found the food already ready, they left me in peace, and let's say that I was lucky at this point. I mean, I received support from my family, and that's why I didn't want to leave because they had also offered some houses, some places for us nurses to stay during this period; I didn't want to go because I needed my family, I needed (...) to arrive home and find all these things.” (I-P23)*

“*My partner's smile, my partner's smile, yes. He understood what my job was, a lot. He was close to me and everything.” (I-P8)*

Talking about the stressful and burdensome situations at the workplace with family members or close friends was a frequently applied strategy to cope with psychologically demanding circumstances.

It was interesting to hear what some of the participants, who are mothers, reported about the obstacles they faced during the COVID-19 pandemic, not only at work but also in the family, since they did not have time to take care of the children, who in many cases stayed at home alone. One of them pointed out.

“*How do I manage with the children? When the schools closed, my husband also had to work. That was a big challenge. Um, I wrote a diary myself as a coping strategy, which has proven quite good. Then I tried. At some point, I stopped watching the news (...) because it drove me crazy.” (A-P25)*

## Discussion

This study describes the challenges of nurses and nursing assistants with the novel disease while caring for COVID-19 patients during the first wave of the pandemic in Austria, Germany, and Italy. The main findings of our qualitative analysis demonstrated that participants expressed their experiences on three themes: Knowledge, skills, and training; resources and risks for health care workers; and coping strategies.

The unknown course and treatment of the virus put pressure, fear, and uncertainty on the nurses and nursing assistants interviewed, especially at the beginning of the pandemic, which they perceived as a “tsunami” situation. Accordingly, Arcadi et al. (14) found that healthcare professionals were confronted with an unknown disease without knowing about it and being prepared for it. Facing the emergent situation, the facilities worldwide were unprepared for this pandemic, and hospitals could not admit patients who were dying rapidly. Similarly to the participants of Levi and Moss's (33) study, our participants experienced many changes in practice, including work overload, lack of or constant changes in knowledge about the COVID-19 virus and hospital policies, the inability to provide standard quality care to patients, and vanity. Under these circumstances, there was only erratic, unconscious, inconsistent, and limited information about the treatment and management of this novel disease.

It has been demonstrated that there is high individual-level heterogeneity in willingness to comply with COVID-19 preventive behaviors such as mask-wearing and hand hygiene among the general population as well as among healthcare workers (34, 35). Uncertainty and limited information are particularly problematic as they can make healthcare professionals prone to misinformation, which is in turn associated with noncompliance with preventive behaviors (35), reduce the quality of care, and increase their fear, anxiety, and stress (36). Thus, it is crucial to not only communicate changes in guidelines to the healthcare professionals but also to explain the identifying evidence that led to the updated guidelines. Our findings support previous studies that reported organizational problems related to a lack of personal protective equipment (PPE), resources, personnel, or training (10, 14, 37). Many of this study participants mentioned “Learning by doing.” They indicated that they had learned how to care for isolated patients in COVID-19 clinics, perform isolation procedures, use PPE, and understand the importance of communication and engagement.

The speed of the spread of COVID-19 made it extremely important for healthcare professionals worldwide to use PPE. Countries generally unprepared and most affected by the pandemic experienced problems accessing PPE. This study found that participants from Italy did not have enough PPE supplies, particularly at the beginning, while in Germany and Austria, it was reported to have been misused. In addition, the requirement to use PPE for extended periods led to deviations from the proper use of PPE recommended in the guidelines and various other problems (33). According to Akkus et al. (8), nurses experienced issues with sweating, headaches, redness and irritation of facial skin, and dry mouth while wearing PPE. The current study also found that wearing PPE by nurses and nurse assistants resulted in physical problems, additional workload, and time applying dressings (9, 10, 38). Chen et al. (39) highlighted that difficulties associated with using PPE also affected hearing and communication. The masks cover lip movements and facial expressions, and the visor over the head protects the ears. Consequently, this leads to problems between patients and healthcare professionals (8). Modern technologies and the production of PPE that is more sound permeable and makes lip movements visible can be helpful in clinical practice.

Although others in the community had to stay home and avoid social contact, healthcare professionals, especially nurses and nursing assistants, continued to be exposed to the virus while providing care (8, 10, 40). They reported fear of infection and infecting their families, financial pressures, long working hours without adequate food, lack of PPE, death of patients and colleagues, and lack of proper child supervision. Self-quarantine, isolation, children's decline in education, fear regarding the future, PPE issues, and the feeling of hopelessness in caring for patients additionally caused emotional burdens for our participants during this outbreak. This finding is consistent with and supportive of other studies about nurses and nursing assistants working with COVID-19 patients. Previous studies have also yielded similar results (8, 38, 41). In their study, Moore et al. (40) found that healthcare professionals fear uncertainty for themselves and their family's health but are willing to take on the risk of caring for patients. Furthermore, similarly to our findings, Morgan et al. (36) reported that women healthcare professionals who had children were susceptible to stress, anxiety, and depression during COVID-19. To overcome the mentioned issues, monitoring nurses' psychological problems and implementing early interventions such as professional psychological counseling will be helpful (8, 40, 41).

While the present analyses mainly focused on nurses and nursing experiences during the first phase of the pandemic, it does not emphasize the mental issues caused by the long-lasting overload. The Chen et al. (12) meta-analysis is interesting in this context, which found that the high rate of symptoms such as anxiety, depression, distress, or insomnia strongly affected the general population and healthcare professionals. Despite the difficult and stressful situation they were living, nurses and nursing assistants who participated in this study attempted to cope with their experiences. Therefore, coping strategies are essential for handling the COVID-19 pandemic. Despite the critical situation, analyses carried out by many researchers worldwide during the COVID-19 pandemic show that nurses have enough resources to employ constructive coping strategies, which are very important for nurses and nursing assistants to handle pandemic situations (41, 42).

The three main themes (and their subcategories) identified in our study (i) knowledge, skills, and training; (ii) resources and risks for the healthcare professionals; and (iii) coping strategies are related to the healthcare workers' organizational support, and the failure of support and coping can lead to mental health issues among healthcare professionals as demonstrated previously. Organizational support, in particular work support (e.g., access to appropriate PPE; rapid availability of COVID-19 tests) and personal support (e.g., access to childcare; provision of food, lodging, transportation), has been shown to predict different levels of anxiety and levels of life (dis)satisfaction during the COVID-19 pandemic (43). Access to PPE predicted better physical health, job satisfaction, and lower distress in healthcare workers. Those unsure about their COVID-19 infection status were more likely to experience distress, anxiety, and depression symptoms and had lower job satisfaction (18, 44). Findings from Peru indicated that healthcare workers with a lower education level were more anxious, and younger healthcare workers and those in the private sector were more susceptible to job turnover (45).

Interestingly, the chance of having distress and depression symptoms varied significantly by the number of working days among younger healthcare workers and were negatively associated with job satisfaction and life satisfaction but positively associated with job turnover intention. In contrast, office days were positively associated with job and life satisfaction among older colleagues, suggesting that younger healthcare staff who did not work many days and senior healthcare staff who worked more days are the most mentally vulnerable groups (44, 46). As revealed by the meta-regression analysis by Chen et al. (12), frontline healthcare workers suffered more from mental symptoms than general healthcare workers, and PTSD had the highest prevalence rates in both groups among all mental health symptoms examined.

The results of the present study, similar to Zhang et al. (18) findings, show that nurses and nursing assistants used different coping strategies to manage the difficulties of working in wards with high COVID-19 mortality. In addition to their challenges, our study participants positively took measures to cope with the situation and their emotions, managing to adapt to the problem by themselves. They got new information online, followed infection control procedures, and used appropriate PPE and protection techniques to reduce stress. Literature data also show that access to reliable information is the most common way of reducing adverse consequences of distress and maintaining a sense of security (39, 42, 47). Similar to previous studies, our study participants used positive attitudes, clear hospital policies, and infection prevention knowledge to reduce team stress during the COVID-19 outbreak, helped their colleagues by teaching them updated skills and techniques, and provided a team approach (16, 41, 48).

Similarly, participants in the Lee et al. study thought that positive thinking and a friendly work environment reduced stress when caring for COVID-19 patients (49). In addition, participants relied on using self-care and support as an adopting strategy. They communicated among themselves, with family members and other friends who listened without judgment and provided their support.

Furthermore, several participants of this study developed new strategies and rituals for taking care of themselves (11, 42). All participants described that a sense of companionship supported them among colleagues working together and sharing jokes or humor with colleagues and patients. Accordingly, Rose et al. (15) reported that humor in the workplace, transparent and frequent communication, and PPE availability were among the coping methods health care workers used to combat the ongoing pandemic. Arcardi et al. (14) reported that healthcare professionals showed solidarity and team spirit in their study.

During the pandemic, the experience and skills of nurses and nursing assistants improved, and they had the chance to strengthen their skills by participating in a multidisciplinary team (10). Similarly, our study participants said they helped each other by sharing and acquiring knowledge, reinforcing their knowledge base, and finding new solutions to the current situation (6). Although the situation has improved, institutional administrators need to advance PPE- and pandemic-related education, including scenarios such as the COVID-19 pandemic, to increase and support the preparedness of nurses and nursing assistants for future infectious diseases (39). Communication tools should also be improved, as the lack of communication and information was problematic in many cases reported in this study.

Finally, this project showed that a positive attitude and conversations with family, friends, and the team leader were the most important coping strategies among nurses and nursing assistants. As Slama et al. (16) noted in their study, almost all participants described how they sought support from their family and friends. Families were their primary source of support in vulnerable situations, protecting them from or cushioning them against the psychological impact of the pandemic (18, 49). Similarly, Pulido-Fuentes et al. (50) demonstrated the critical role of primary care family and community health services in protecting emotional health and maintaining mental health, even among direct care workers. Family and friends' support and positive attitudes have been shown to reduce perceived stress and be more supportive during infectious disease outbreaks (16). We found similar results in our study. A supportive attitude and communication with family and friends were crucial elements in coping reported by our participants. Although these study participants relied on self-care coping strategies, several described developing new strategies and rituals for taking care of themselves. Similar qualitative evidence uncovered healthcare professionals' self-strategies to deal with difficult situations (11, 42, 47, 51). These strategies help nurses and nursing assistants to become more professional and inspire them in mindfulness.

During the interviews, the participants addressed various other aspects of the pandemic, including ethical and legal issues, organizational influences, (interprofessional) teamwork, impact on the social environment, long-term impacts of the pandemic, and mental and emotional reactions. These aspects will be addressed in subsequent publications, where mental health will be discussed in-depth.

## Conclusion

This study examined the difficulties nurses and nursing assistants experienced in caring for COVID-19 patients in hospitals and nursing homes in Austria, Germany, and Italy. They presented many challenges, including lack of knowledge about an unknown disease, organizational problems such as lack of staff and PPE, training problems, difficulties in using PPE, risk and infection, isolation, and different feelings. To overcome these challenges, participants in this study used different coping strategies, such as humor and laughing with each other and with patients, adapting to the new situation and learning to cope with reality, creating warm teamwork, receiving and providing support, taking care of themselves, and especially communicating and being supported by family and friends. Therefore, some of these strategies can be applied to reduce these challenges and create better working conditions for nurses and nursing assistants in similar events. These include providing the proper and sufficient PPE, a good work environment, financial and social support, managing pandemic situations effectively, developing new ways of communication between staff, patients, and families, and paying more attention to healthcare professionals. Further research, training of staff, and adaptation of institutional policies may help develop new strategies to combat future pandemics successfully.

## Strengths and limitations

The results of our research may influence the improvement of nursing care and management during pandemics. It is a contributing factor to the literature on healthcare during a pandemic. This study provides information on the challenges nurses, and nursing assistants lived during the COVID-19 pandemic in different settings (hospitals, nursing homes) in three European countries. Most of our sample are nurses, as they are the healthcare professionals with the most contact with patients in the studied settings. Our findings are limited to nurses and nursing assistants who worked during the COVID-19 pandemic in Austria, Germany, and Italy.

Furthermore, the travel restriction made it impossible to conduct the interviews face-to-face. Still, they had to be completed online *via* Zoom, which might be considered a limitation. Future research should examine health professionals' difficulties during COVID-19 in different countries, including non-EU countries.

## Data availability statement

Data are not publicly deposited but are available (Health University of Applied Sciences Tyrol, Austria) upon request to the authors.

## Ethics statement

The studies involving human participants were reviewed and approved by RCSEQ. The patients/participants provided their written informed consent to participate in this study.

## Author contributions

NP, SP, and CZ contributed to conception and design of the study. Data extraction: NP, CR, and BB. Analysis: NP, CR, and CZ. NP led and wrote the first draft of the manuscript, with the contribution of CZ. All authors contributed to manuscript revision, read, and approved the submitted version.

## Conflict of interest

The authors declare that the research was conducted in the absence of any commercial or financial relationships that could be construed as a potential conflict of interest.

## Publisher's note

All claims expressed in this article are solely those of the authors and do not necessarily represent those of their affiliated organizations, or those of the publisher, the editors and the reviewers. Any product that may be evaluated in this article, or claim that may be made by its manufacturer, is not guaranteed or endorsed by the publisher.
